# *Bartonella henselae* endocarditis in an elderly patient

**DOI:** 10.1371/journal.pntd.0008376

**Published:** 2020-07-30

**Authors:** Marina Rovani Drummond, Amanda Roberta de Almeida, Letícia Valandro, Maria Helena Postal Pavan, Raquel Silveira Bello Stucchi, Francisco Hideo Aoki, Paulo Eduardo Neves Ferreira Velho

**Affiliations:** 1 Applied Research in Dermatology and Bartonella Infection Laboratory, University of Campinas (UNICAMP) Medical School, Campinas, Sao Paulo, Brazil; 2 Division of Dermatology, Department of Medicine, University of Campinas (UNICAMP) Medical School, Campinas, Sao Paulo, Brazil; 3 Division of Infectious Diseases, Department of Medicine, University of Campinas (UNICAMP) Medical School, Campinas, Sao Paulo, Brazil; Yale University School of Medicine, UNITED STATES

## Summary

We report an 85-year-old white man admitted to the emergency department of the University of Campinas with fever of undetermined origin (FUO) who received antibiotics previously. Initially, the hypothesis was pneumonia. He presented a drug reaction misdiagnosed as staphylococcal desquamation. The follow-up confirmed that prolonged fever was caused by bacterial endocarditis by transthoracic echocardiogram that showed vegetation in the aortic valve. *Bartonella henselae* etiology was confirmed by PCR.

This case reinforces the difficulty of diagnosing *Bartonella* sp. infection; this etiology must be considered even in patients with negative serology. The criteria for the diagnosis of bacterial endocarditis should contemplate a molecular positivity investigation for *Bartonella* spp, such as PCR in blood or serum samples as a major Duke criterion, even if with titers lower than 1 to 800.

## Presentation of case

An 85-year-old white man was admitted to the University of Campinas Hospital (22° 54′ 21′′ S; 47° 3′ 39′′ W), Brazil, presenting a fifteen-day history of daily fever and pruritic scaly lesions on his trunk, limbs, palms, and cervical region, along with oral-mucosa ulcers. Two months earlier, he was diagnosed with pneumonia and was treated with antibiotics; the names or duration are unknown. He denied comorbidities, smoking, and alcoholism. He occasionally used dipyrone due to pain or fever. He reported daily contact with dogs.

The patient had 38.5°C fever, erythematous-papular-scaly lesions on the mentioned areas. He presented lamellar desquamation on palms and enanthema with ulcerations in the back of the tongue and lips. The spleen was percutible, and the liver palpable was 3 cm from the right costal border. He presented no other alteration, including cardiac auscultation.

The initial exams showed hemoglobin of 12.5 g/dL, leucopenia of 2,600 cells/mm^3^, and thrombocytopenia of 97,000 cells/ml of blood. Chest X-ray was normal. Abdominal ultrasound showed alteration in liver texture (but normal volume) and homogeneous splenomegaly (15.6 cm on the largest axis).

After collecting cultures, he was treated with clindamycin for staphylococcal maculopapular desquamation. The patient became afebrile after 24 hours and evolved with confluence of papules forming extensive plaques in the thighs.

The next day, he was reevaluated. Palmar lamellar desquamation strongly suggested FUO associated with drug reaction. The antibiotic and dipyrone were suspended, and daily oral use of 40 mg prednisone was initiated.

Fever reoccurred. On subsequent days, skin lesions improved, and corticotherapy was suspended. The patient maintained a daily fever and had frequent episodes of dyspnea, wheezing, and coughs, but remained hemodynamically stable. Three blood and two urinary diagnostic routine culture results were negative. The chest and abdomen CT scans showed no lymph node enlargement. Serological tests for syphilis, hepatitis B and C, HIV, mononucleosis, and toxoplasmosis showed no infection. Immunofluorescence assays (IFA) for *B*. *henselae* and *B*. *quintana* were more than 1 to 64. During the investigation, *Bartonella* sp. cultures and molecular research were requested from a blood sample; a transthoracic echocardiogram showed aortic valve with mild thickening of a noncoronary leaflet, with a pedunculated image of 7x2 mm. The other valves had normal morphology and mobility.

Once the diagnosis of infectious endocarditis (IE) was confirmed, intravenous therapy with gentamicin and ampicillin and sulbactam (AS) was initiated, improving respiratory and feverish conditions.

After 42 days of AS treatment, the patient’s image of vegetation remained unchanged, but surgical intervention was dismissed, based on his clinical status.

Molecular tests targeting *Bartonella* spp. DNA was performed from whole blood (collected at the same day of *Bartonella* sp. serology) and liquid culture. *B*. *henselae* DNA was detected by nested PCR targeting *fstZ* region in both samples [1]. Subsequently, amplicons were sequenced and confirmed to be 100% homologous to *B*. *henselae* Houston-1 (access number CP020742.1). The conventional PCR targeting *ITS* region, genera-specific, was performed and was negative. The sensitivity of the conventional PCR was 50 genomic copies, and the nested PCR was 10 genomic copies, with no isolate in the solid culture. In order to test carryover contamination, we added negative controls at different stages of the PCR process, as previously described, and all remained negative throughout procedures [2].

Upon discharge, the medical team prescribed 200 mg doxycycline per os daily for six weeks, having received a *B*. *henselae–*DNA positive detection from the patient's blood sample.

The patient had a good clinical evolution; five years later, he had no complications related to endocardial vegetation.

The patient in this manuscript has given written informed consent (as outlined in the PLOS consent form) to publication of his case details.

## Case discussion

Older adults have higher risk of IE than the general population [3, 4]. The incidence of IE in this population increases with age, and the prevalence of degenerative valve lesions [5]. Their disease is more often atypical [5, 6]. Fever may not happen, delaying diagnosis and increasing mortality [5]. Dyspnea can be explained by the deterioration of cardiac function [7].

Initially, the hypothesis of this elderly patient with FUO was pneumonia, and he received antibiotics. The follow-up confirmed IE.

The use of antibiotics prior to culture collection is considered the main cause of negative diagnostic routine cultures of IE, hindering growth, even under appropriate conditions, of fastidious agents, such as *B*. *henselae* [8, 9]. Thus, the use of molecular methods enables the etiological diagnosis of endocarditis previously considered idiopathic [10, 11].

Previously, physicians believed that *B*. *henselae* growth was only possible in cell cocultures. Subsequently, it was possible to get primary isolation of gram-negative coccobacilli up to 6 weeks after sowing in solid medium enriched with blood (5% to 30%), microaerophilic environment, and temperature between 35°C to 37°C [12, 13]. Liquid enrichment cultures increase the possibility of bacterium isolation [14].

In addition to being extremely demanding bacteria, the diagnosis of IE by these agents can be difficult, even in patients with bloodstream infections who did not use antibiotics since false-negative results from PCRs are described in blood and liquid culture [15, 16]. The sensitivity of the method (conventional or nested PCR) is another variable in false-negative results [13, 17, 18].

Besides difficulties of molecular tests, serology is not considered a gold standard for diagnosis of infection. Serology more than 1 to 800 has been used as a major criterion for the diagnosis of IE, but there are reported cases of the disease with serology with lower titers [11]. On the other hand, false-negative tests have been described with different strains of *B*. *henselae* and false-positive reactions with other species of the same genus and other bacteria [12, 19–22].

In spite of negative diagnostic results, the clinical presentation of endocarditis caused by *Bartonella* sp. must be valued. Splenomegaly and aortic valve involvement are frequent, typically producing noninvasive IE [12], as in this case.

This report reinforces the diagnostic difficulty of *Bartonella* sp. infection and that this etiology must be considered even in patients with negative serology, reinforcing the opinion of Spach and colleagues [23] that the criteria for diagnosis of IE should contemplate a positivity of the molecular investigation for *Bartonella* spp. in blood or serum samples as a major Duke criterion, even with titers lower than 1 to 800.

## Key learning points

- Older adults have higher risk of IE than the general population.
- *Bartonella* sp. laboratory diagnosis is difficult, and infection must be considered even in patients with negative serology.
- *Bartonella* sp. serology titers lower than 1 to 800 do not exclude this etiology.
- Diagnosis of bacterial endocarditis should contemplate molecular positivity investigation for *Bartonella* spp. such as PCR in blood or serum samples as a major Duke criterion.
